# Supplementary material for: Patterns of Intron Gain and Loss in Fungi
Source: PLoS Biol. 2004 Nov 30;2(12):e422. doi: 10.1371/journal.pbio.0020422 (PMC532390; doi:10.1371/journal.pbio.0020422)
Supplement: Table S1 — Also available at http://genes.mit.edu/NielsenEtAl/. (4.3 MB ZIP). [file pbio.0020422.st001.zip › NielsenEtAl/html/1272.html]

AN3789.1.NCU02459.1.MG04091.1.FG00949.1


```
 CLUSTAL W (1.82) Multiple Sequence Alignments - Introns Inserted


Sequence 1: NCU02459.1	355 aa
Sequence 2: FG00949.1	390 aa
Sequence 3: MG04091.1	338 aa
Sequence 4: AN3789.1	366 aa
Alignment Length: 465 aa
Number Identitical Residues: 107 aa
Alignment Score (without introns) 5770


MG04091.1 	----------------MSGIRRASHAGSWYTHSSSKLNTELDEWLSEVPDKINES-DVPI
NCU02459.1	MEAAARDWSKSDGHGDFQRTRPASHAGSWYLGNAARLSSQLDEFMSRVPNKLDGR-DLPI
FG00949.1 	-----------------MATRPAKKAGYWYKSEAAELKSELQQYMAAVPESINGA-PVPV
AN3789.1  	-----------------MGFRAASHAGSWYSGNRQTLALQLDQWLDRVPGTLEGLGSLPA
          	                    * *.:** **  .   *  :*::::  ** .::  . :* 

MG04091.1 	KGASVIIAP2HAGYSYSGPCAAWAYKVFDVSAA~KRIFVLGPSHTYYLSGCALTTYATYE
NCU02459.1	PGARVIIAP2HAGYSYSGPCAAWAYKILDLANV~KRVFLLGPSHTFYLKGCALSTFGKYS
FG00949.1 	PGARVVIAP2HAGYEYSGPCAAWAYKTLDLSCA~KRVFVLGPSHTYYLEGCAATIFGKYA
AN3789.1  	PGARIIIAP2HAGYSYSGPCAAYAYKALDLSKA2KRIFVLGPSHHHYFTSLALPTLTGYY
          	 ** ::*** ****.*******:*** :*:: . **:*:***** .*: . * .    * 

MG04091.1 	TPLGN--LRVDLDTIKQLRDT---------GKFKDIPRDNDEDEHSLEMHLPYLAKRLTQ
NCU02459.1	TPFGD--LVVDGKAVDELMED---------QKFSPIPVEYDIREHCLEMHLPYLWKRLEQ
FG00949.1 	TPFGD--LEIDVDMAKELEDA---------IMMEKMPRQGEINEHSLEMHMPYLYLRCEE
AN3789.1  	TPLSDDPLPLDTELIAKLRSTPAAKSNGSTIAFEDMSQSVDEDEHSIELHLPYIHRLLQL
          	**:.:..* :* .   :* . .::.:..::  :. :. . :  **.:*:*:**:      

MG04091.1 	TFGGGSDGDGDASWPPVVPILIGDNKRDAEKAFGELLLPHLRDPDNAFIVSSDFCHWGNR
NCU02459.1	TLGGD-----SSQFPPIVPVLVGDLSADGEKAVGSILAPYLADPKNAFIISSDFCHWGKN
FG00949.1 	TFET------PDKFPKIVPVLVGSNTAKEEKVIGRALLPYLRDPENAFIISSDFCHWGSG
AN3789.1  	QHPDKK----AAEYPPLVPILVGSTSKATEAAFGALLAPYLEDPSNAFVVSSDFCHWGLR
          	     .      .:* :**:*:*. .   * ..*  * *:* **.***::********  

MG04091.1 	FSYTKYTADG----TVEGVRSLGRAD----------------------RNLP------VP
NCU02459.1	YHYRPQLHKGLVRDQDAQWASLKIQPG-----------------FKTKADGSEPRDAEVP
FG00949.1 	FSYLPYSPT----NSPSDLTQLKKRD--------------------PKPDGP-------P
AN3789.1  	FRYTYYVPQAPVPGPRLPLSSYDLPQPEDDETEIESKIEVACAGYHLKRRDPIRR-GKPL
          	: *      .   .      .      ....:. .:. . :.:.   .   .    ..  

MG04091.1 	IHEGIRVLDHLAMDAIETGSHDAFYDNLKATGNTVCGRHPIGVVMAALEMLKKERAGGDG
NCU02459.1	IHEVIKALDDLVMDSIKTGDHSDYYSILKGTNNTVCGRHPIGVVLAALEKMGG-------
FG00949.1 	IHETIRVIDQAAMDAVETGSHDAFISTLKQTRNTVCGRHPIGVTMAALELLQK-------
AN3789.1  	IHESISSFDIATMAAIATGKTERFTDILEKTGNTVCGRHPIGVIMAAIEAAGK-------
          	*** *  :*  .* :: **. . : . *: * *********** :**:*           

MG04091.1 	AASGSKKGDGVFTFVKYDRSSLVEN-----~-------VSDSSVSYAAAYAVI--~----
NCU02459.1	AQSGESKGK--FQFVQYQRSNLVE------~------KQSDFSVSYVSAYSIL--~----
FG00949.1 	EAGFEEKGR--FSIIQYNRSNMVEEPNGMS0YFRSQPRRSPLETELVECQLVMRS1HEQV
AN3789.1  	QEGG--KGK--FHFARYERSSDIGE-----~------IVEDSSVSYVSAFAVV--~----
          	  .   **   * : :*:**. : .              .  ... . .  ::       

MG04091.1 	-------------------------------------------------
NCU02459.1	-------------------------------------------------
FG00949.1 	IGFPWQCRYEPLLTHAAKLNLGSFETPPLSLSLKWVDAIFAELRLTPTN
AN3789.1  	-------------------------------------------------
          	
```
